# PRISM-games 3.0: Stochastic Game Verification with Concurrency, Equilibria and Time

**DOI:** 10.1007/978-3-030-53291-8_25

**Published:** 2020-06-16

**Authors:** Marta Kwiatkowska, Gethin Norman, David Parker, Gabriel Santos

**Affiliations:** 8 Microsoft Research Lab, Redmond, WA USA; 9 University of Southern California, Los Angeles, CA USA; 10 Department of Computing Science, University of Oxford, Oxford, UK; 11 School of Computing Science, University of Glasgow, Glasgow, UK; 12 School of Computer Science, University of Birmingham, Birmingham, UK

## Abstract

We present a major new release of the PRISM-games model checker, featuring multiple significant advances in its support for verification and strategy synthesis of stochastic games. Firstly, *concurrent* stochastic games bring more realistic modelling of agents interacting in a concurrent fashion. Secondly, *equilibria*-based properties provide a means to analyse games in which competing or collaborating players are driven by distinct objectives. Thirdly, a *real-time* extension of (turn-based) stochastic games facilitates verification and strategy synthesis for systems where timing is a crucial aspect. This paper describes the advances made in the tool’s modelling language, property specification language and model checking engines in order to implement this new functionality. We also summarise the performance and scalability of the tool, and describe a selection of case studies, ranging from security protocols to robot coordination, which highlight the benefits of the new features.

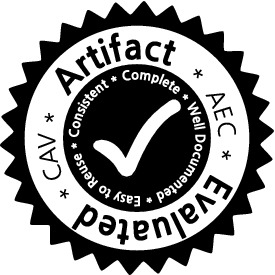



## Introduction

Quantitative verification and strategy synthesis are powerful techniques for the modelling and analysis of computerised systems which require reasoning about *quantitative* aspects such as probability, time or resource usage. They can be used either to produce formal *guarantees* about a system’s behaviour, for example relating to its safety, reliability or efficiency, or to synthesise controllers which ensure that such guarantees will be met at runtime. Examples of applications where these techniques have been used include power controllers, unmanned aerial vehicles, autonomous driving and communication protocols.

As computing systems increasingly involve concurrently acting autonomous agents, *game-theoretic* approaches are becoming widespread in computer science as a faithful modelling abstraction. These techniques can be used to reason about the *competitive* or *collaborative* behaviour of multiple rational agents or entities with distinct goals or objectives. Applications include designing a defence strategy against attackers in a cybersecurity context or building controllers for autonomous robots operating in an unknown or potentially malicious environment. More broadly, game theory techniques such as mechanism design can be used to design protocols that are robust in the context of selfish participants, for example by incorporating *incentive/reward* schemes. They have been successfully deployed in diverse contexts such as network routing [29], auction design [10], public good provisioning [15] and ranking or recommender systems [30].

However, designing game-theoretic systems correctly is a challenge, in view of the complexity of behaviours arising from the interactions between autonomy, concurrency and quantitative rewards. This motivates the development of formal verification techniques to check their correctness and synthesise correct-by-construction strategies for them. Furthermore, many of these applications require reasoning about *stochasticity*: protocols may employ randomisation, e.g., for reliable dissemination across a network, or to minimise the impact of information leakage to an observer; autonomous robots operate in uncertain environments and may use unreliable hardware components or noisy sensors; and data-driven systems such as ranking or navigation systems rely on learnt probabilistic models for their execution.

These challenges have inspired the development of PRISM-games [22], a model checking tool for *stochastic games*. To date, it supports verification and strategy synthesis for *turn-based* stochastic multi-player games (TSGs) using a variety of objectives, expressed in the temporal logic rPATL (probabilistic alternating-time temporal logic with rewards) [8]. This allows specification of *zero-sum* objectives relating to one coalition of players trying to maximise a probabilistic or reward-based objective, while the remaining players form a second coalition trying to minimise the objective. It has also been extended to include (zero-sum) *multi-objective* properties and additional reward measures such as *long-run average* and *ratio reward* [22]. These methods have been successfully applied to several case studies such as autonomous vehicles, user-centric networks, temperature control and an aircraft electric power system [21, 23, 32].

In this paper, we present PRISM-games 3.0, which significantly extends its predecessor’s functionality in several ways [18–20]. First, it supports the modelling and analysis of *concurrent stochastic multi-player games* (CSGs). Previous versions of the tool supported TSGs, in which it is assumed that each state of the game is controlled by a specific player. CSGs allow players to make decisions simultaneously, without knowledge of each other’s choices, providing a more realistic model of concurrent execution and decision making. For this, we extend the PRISM-games modelling language, allowing the user to specify concurrency and synchronisation among agents, as well as to associate rewards to either joint or single actions.

In the first instance, PRISM-games now supports verification and strategy synthesis for CSGs using zero-sum specifications in rPATL [19], which we extend to accommodate *instantaneous rewards*. The second major addition to the tool is the possibility of reasoning about *equilibria-based* properties, which allow players to have distinct, not necessarily conflicting objectives. We extend rPATL to express properties relating to (subgame perfect) *social-welfare optimal Nash equilibria (SWNE)* [20]. This provides synthesis of strategies for all players (or coalitions) from which there is no incentive for any of them to unilaterally deviate in any state of the game, and where the combined probabilities or rewards are maximised (or minimised).

Thirdly, PRISM-games now adds support for *probabilistic timed multi-player games* (TPTGs) [18] (currently just the turn-based variant of the model). These extend stochastic multi-player games with real-valued clocks, in the style of (probabilistic) timed automata. This allows real-time aspects of a system to be more accurately modelled. Using the *digital clocks* approach [18], timed models are automatically translated to discrete-time models in order to be verified.

In this paper, we describe the key enhancements made to the tool, notably to its modelling and property specification languages. We also summarise the results, algorithms and implementation of the verification and strategy synthesis techniques developed [18–20] to support the new functionality. We then describe a selection of case studies which showcase the advantages of the new features, and summarise the performance and scalability of the tool.

PRISM-games is open source and runs on all major operating systems. It is available from the tool’s website [34]. Supporting material for the paper, including a virtual machine that allows easy running of the tool and reproduction of the results presented in Sect. 4, can be found at [33].

**Related Tools.** Other model checking tools have been developed to provide support for games. For non-stochastic games, model checking tools such as PRALINE [5], EAGLE [31] and EVE [16] support Nash equilibria [27], as does MCMAS-SLK [6] via strategy logic. Uppaal Stratego [11] is a tool that uses machine learning, model checking and simulation for the synthesis of strategies for stochastic priced timed games. GAVS+ [9] is a general-purpose tool for algorithmic game solving, supporting TSGs and (non-stochastic) concurrent games, but not CSGs. GIST [7] allows the analysis of $\omega$-regular properties on probabilistic games, but again focuses on turn-based, not concurrent, games. General purpose tools such as Gambit [26] can compute a variety of equilibria but not for stochastic games.

## Modelling and Property Specification Languages

### Modelling Concurrent and Timed Games

The new features in PRISM-games 3.0 have required some significant enhancements to the language used to specify models. For the addition of real-time aspects (i.e., TPTGs), the changes are a straightforward combination of the existing language features for specifying TSGs in PRISM-games (player specifications and mapping of model states to them) and for probabilistic timed automata in PRISM (clock variables, module invariants, guards and clock resets). We therefore focus in this paper on the specification of CSGs, where the language changes are more fundamental.

PRISM-games has an existing language for specifying TSGs, which is an extension of the native PRISM modelling language [22]. Components of the system to be modelled are encapsulated as *modules*, whose states are defined by a set of finite-range *variables* and whose behaviour is specified using action-labelled *guarded commands*. In a state, one or more modules can execute a command to make a transition: if the guard (a predicate over state variables) is satisfied, the state can be modified (probabilistically) by applying the *updates* of the command. Multiple modules can execute simultaneously if their commands are labelled with the same action.

**Fig. 1.**
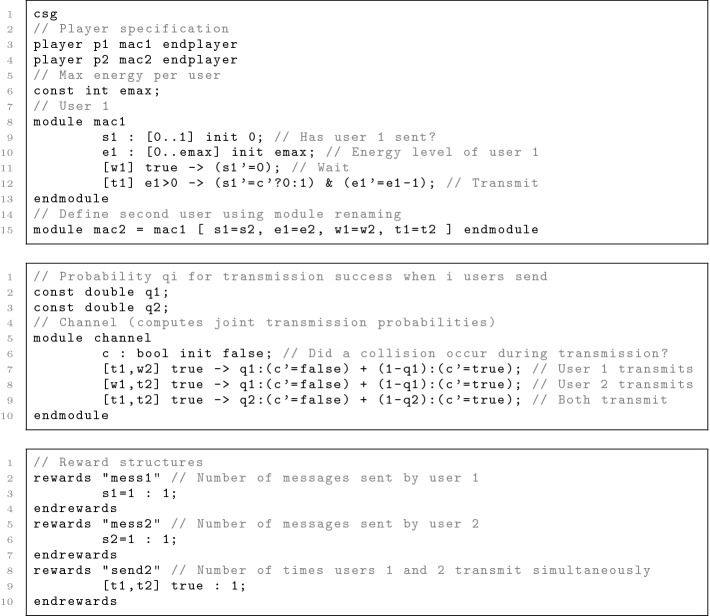
An example PRISM-games 3.0 CSG model of medium access control.

CSGs cannot naturally be modelled with this approach for several reasons: (i) players need to be able to concurrently choose between multiple commands with different action labels; (ii) the update performed by one player may be different depending on the action chosen by another player; (iii) when multiple players execute, variables may need to be updated according to an arbitrary probability distribution, rather than being limited to the product of separate distributions specified locally by individual modules.

Figure 1 shows an example of the PRISM-games 3.0 modelling language, which we use to illustrate some of its new features. It models a probabilistic version of the *medium access control* problem, previously described in [5]. Two users share a communication channel. At each time step, user maci (i = 1, 2) can choose between transmitting a message (ti) or waiting (wi). Variable si tracks whether a user successfully sent its message in the last time step and ei represents its energy level: transmissions can only occur when energy is positive. A third component is the channel channel, modelled by Boolean variable c denoting whether a collision occurred on the last transmission attempt.

The first difference (with respect to modelling of TSGs) is the player specification: players are associated with modules (rather than states). In the example, module maci constitutes player i. Modules with no nondeterministic choice (like channel) do not need to be tied to a player.

In each state of the CSG, each player chooses between enabled commands of the corresponding modules; if no command is enabled, the player idles. The players move simultaneously so transitions are labelled with *lists* of action labels ${[}{} \texttt {a}_1,\dots ,\texttt {a}_n{]}$. So the guarded command notation is extended accordingly: note how the channel’s behaviour depends on which actions the two users take (the same principle applies when specifying reward structures; see send2). Furthermore, variable updates within a command can now be dependent on the updated values of other variables, provided there are no cyclic dependencies. See for example (s1’=c’?0:1), which updates s1 depending on whether there was a channel collision (reflected in c’, the updated value of c). We use this mechanism to model interference on the channel: module channel specifies a joint probability distribution which is used to update variables s1 and s2 simultaneously.

### Property Specification

PRISM-games 3.0 also extends the language used to specify properties for verification and strategy synthesis. The previous version already supported *zero-sum* queries for TSGs using the logic rPATL, which combines the game logic ATL with reward-based extensions of the probabilistic logic PCTL. Again, for the new real-time models, it is relatively easy to combine the existing rPATL notation with real-valued time bounds. So, we focus here on the case of CSGs, and in particular *equilibria-based* properties.

We compute values or synthesise strategies which are *social-welfare optimal Nash equilibria (SWNE)*, i.e., which maximise (or minimise) the sum of the values associated to the objectives for each player, but from which there is no incentive for any of them to unilaterally deviate in any state of the game. We express such properties by adding to rPATL the $+$ operator, which is then used to denote the sum of the values associated to both *bounded* and *unbounded* objectives.

When using the rewards operator in equilibria-based properties, we can reason about *cumulative* ($\mathtt {C}^{\leqslant \mathtt {k}}$), *instantaneous* ($\mathtt {I}^{= \mathtt {k}}$) and *expected reachability* ($\mathtt {F}$) objectives. For properties with the probability operator, we support bounded and unbounded reachability using the temporal operators *next* ($\mathtt {X}$), *eventually* ($\mathtt {F}$) and *until* ($\mathtt {U}$). In order to express zero-sum properties for CSGs, we have implemented all the previous temporal operators for probabilistic queries and a subset of the rPATL operators reported in [8] for reward-based queries, adding to that the instantaneous reward operator.

Finally, following the style of rPATL we separate players into *coalitions* with the syntax $\langle \! \langle {coalition} \rangle \! \rangle$, in order to specify the player or association of players for which we seek to maximise or minimise the values for a given zero-sum property. For equilibria-based properties, given that we maximise/minimise the sum, we use the same operator to separate players in different coalitions using a colon, while players in the same coalition are separated by a comma.

The following are examples of both zero-sum and equilibria-based properties for the medium access CSG model described in Fig. 1.

- $\langle \! \langle {\mathtt {p1}} \rangle \! \rangle {\mathtt P}_{\max =?}[\,{\mathtt {s2}{=}0 {\ \mathtt {U}\ }\mathtt {s1}{=}1}\,]$ – what is the maximum probability user 1 can ensure of being the first to transmit, regardless of the behaviour of user 2?
- $\langle \! \langle {\mathtt {p2}} \rangle \! \rangle {\mathtt R}^{\mathtt {mess2}}_{\geqslant 2.0}[\,{{\mathtt {F}\ }\mathtt {e2}{=}0}\,]$ – can user 2 ensure the expected number of messages it sends before running out of energy is at least 2, whatever user does?
- $\langle \! \langle {\mathtt {p1}{:}\mathtt {p2}} \rangle \! \rangle _{\max \geqslant 2}({\mathtt P}_{}[\,{{\mathtt {F}\ }\mathtt {s1}{=}1}\,]\ {+}\ {\mathtt P}_{}[\,{{\mathtt {F}\ }\mathtt {s2}{=}1}\,])$ – if each user’s objective is to send their packet with the maximum probability, is it possible for them to collaborate and both transmit their packets with probability 1?
- $\langle \! \langle {\mathtt {p1}{:}\mathtt {p2}} \rangle \! \rangle _{\max =?}({\mathtt P}_{}[\,{\mathtt {s2}{=}0 {\ \mathtt {U}\ }\mathtt {s1}{=}1}\,]\ {+}\ {\mathtt P}_{}[\,{\mathtt {s1}{=}0 {\ \mathtt {U}\ }\mathtt {s2}{=}1}\,])$ – what is the sum of SWNE values if each user tries to maximise the probability of being the first to successfully transmit?
- $\langle \! \langle {\mathtt {p1}{:}\mathtt {p2}} \rangle \! \rangle _{\max =?}({\mathtt R}^{\mathtt {mess1}}_{}[\,{{\mathtt {F}\ }\mathtt {e1}{=}0}\,]\ {+}\ {\mathtt R}^{\mathtt {mess2}}_{}[\,{\mathtt {C}^{\leqslant \mathtt {k}}}\,])$ – what is the sum of SWNE values if user 1 tries to maximise the expected number of packets before running out of energy and user 2 maximises the expected number of packets in the first *k* steps?

## Verification and Strategy Synthesis Algorithms

### Zero-Sum Properties for CSGs

When verifying zero-sum properties of CSGs, PRISM-games makes use of the model checking algorithms described in [19], which were based on the methods formulated in [2, 3]. We rely on *value iteration* and classical convergence criteria to approximate/compute the values for all states of the game under study, and on solving a *linear program* to compute a *minimax* strategy at each state. This corresponds to solving a *matrix game*, which represents a *one-shot zero-sum* game for the actions of each player in a state. For unbounded properties, the solutions of the matrix games are used to synthesise an optimal (memoryless and randomised) strategy for each player. Prior to this numerical solution phase, we find and remove the states for which the optimal expected reward values are infinite by using the qualitative algorithms developed in [1].

Our current implementation uses the LPsolve [24] library to solve the matrix games at each state. CSGs are built and stored in a explicit-state fashion using an extension of PRISM’s Java-implemented *explicit* (sparse-matrix based) engine.

### Equilibria-Based Properties for CSGs

For equilibria-based properties of CSGs, PRISM-games implements the methods described in [20]. We rely on value iteration and *backwards induction* to approximate/compute values and synthesise strategies that are SWNE. For unbounded properties, we can only compute values that are $\varepsilon$-Nash equilibria, since Nash equilibria are not guaranteed to exist. At each state, we solve a *bimatrix game*, which is a representation of a *one-shot nonzero-sum* game and is a linear complementarity problem. We solve these games via *labelled polytopes*, finding all equilibria values through an SMT-based implementation, for which we use third-party SMT solvers Z3 [12] and Yices [13]. We make use of a precomputation step of finding and removing *dominated strategies* in order to minimise the number of calls to the solver.

Unlike zero-sum properties, the synthesised strategies for bounded and unbounded equilibria-based properties require (finite) memory. This is needed due to the fact that a player’s choices may change once their objectives have been satisfied. We synthesise strategies by combining the strategy vectors computed for each bimatrix game and the strategy generated by computing optimal values for the MDP resulting from playing the game after either goal has been met. As we use value iteration to approximate values for infinite-horizon properties, we can only synthesise $\varepsilon$-Nash strategy profiles.

### Turn-Based Probabilistic Timed Games

Verification and strategy synthesis of TPTGs relies on the algorithms from [18], which use the *digital clocks* approach that has been a developed for a variety of real-time models. A translation, at the level of the PRISM-games modelling languages, automatically converts the problem of analysing a TPTG into one of solving a (discrete-time) TSG, for which PRISM-games’s existing engines can be used. Time-bounded properties are handled by automatically integrating a timing clock into the model prior to translation. As in the rest of PRISM-games, TSGs are also built and solved using the Java-based *explicit* engine.

## Case Studies and Experimental Results

The features added in PRISM-games 3.0 have been used for over 10 new case studies across a wide range of application domains, including computer security (intrusion detection, radio jamming, non-repudiation), communication protocols (medium access control, Aloha), incentive schemes for cooperative networking, multi-robot navigation problems and processor task scheduling. Details can be found in [18–20] and on the case studies section of the PRISM-games website [35]. Supporting material is at [33]. In this section, we showcase four selected case studies that demonstrate the benefits of the tool’s new functionality. We also include a discussion of the scalability and performance of the tool.

**Future Markets Investor.** This example models two investors playing against the stock market. Investors choose when to invest or to cash in, and the stock market can decide to bar investments at certain points; fluctuations in share values are modelled stochastically. PRISM-games can, for example, synthesise optimal strategies for the two investors to maximise their expected joint profit over time, acting against the stock market which aims to minimise it.

**Fig. 2.**
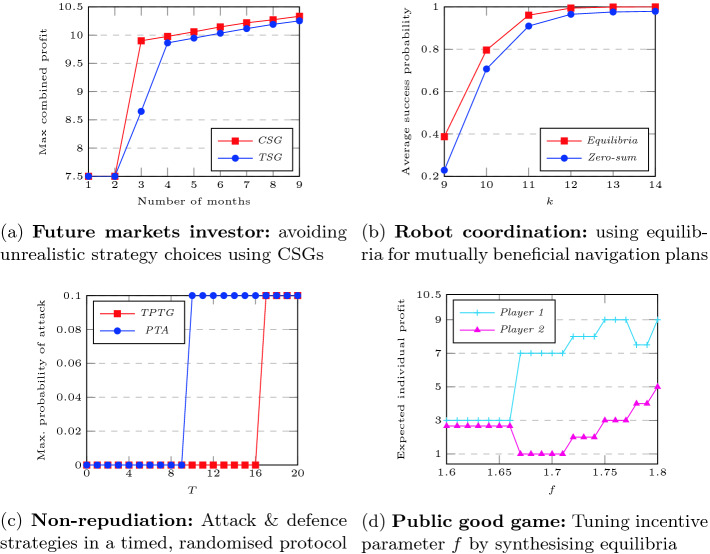
Results illustrating the benefits of the new verification and strategy synthesis techniques implemented in PRISM-games 3.0; see Sect. 4 for details. (Color figure online)

Figure 2(a) shows the results obtained for this property using both a *turn-based* stochastic game (TSG) and a *concurrent* stochastic game (CSG). The former leads to unrealistic modelling as the market can see the choices made by the investors and gain an unfair advantage: the values in the blue plot in Fig. 2(a) are artificially low. In the CSG model, using PRISM 3.0, decisions are taken simultaneously, yielding the correct strategies and values (red plot).

**Robot Coordination.** Our next example models two robots navigating in opposite directions across a 10-by-10 grid as a CSG. Obstacles which hinder the robots as they move from location to location are modelled stochastically; and if the robots collide, both of them fail in their attempt to reach their goal. We use PRISM-games to find navigation strategies for the two robots, where each robot does not know the choice being made by the other at each step.

The objective for each robot is to navigate successfully, so we maximise the average probability (across the two robots) of success. Figure 2(b) shows the best value that can be achieved within a fixed period of *k* moves across the grid. One robot aiming single-handedly to achieve this goal performs reasonably well (blue plot), but we can achieve better collective performance by using PRISM-games to synthesise a (social welfare Nash) *equilibrium* strategy (red plot).

**Non-repudiation.** Next we consider a non-repudiation protocol [25], which permits an originator *O* to transfer information to a recipient *R* while guaranteeing non-repudiation, i.e., that neither *O* nor *R* can deny that they participated in the transfer. Here, both *probability* (the protocol is randomised) and *time* (the protocol relies on acknowledgement time-outs) are essential ingredients for checking correctness. Furthermore, we model the two participants of the protocol as opposing players, resulting in a TPTG model.

To verify the protocol, we check the worst-case probability that a malicious recipient *R* can obtain the information being transferred within time *T*. This can be done with a PTA model (as in [28]) but, with a timed game model, we can also analyse counter-strategies of the honest participant. The results (see Fig. 2(c)) show that, while it is not possible to prevent the information being received, it *is* possible to delay it (the red plot shows lower probabilities for higher times). Note that the bound *T* is an actual time bound, unlike the examples above, where step-bounded properties measure the number of steps or rounds.

**Public Good Game.** Lastly, we show a new case study modelling a *public good game*, a well studied model of social choice in economics where participants repeatedly decide how much of an endowment to keep for themselves or to share it with the other players. The total shared by the players is boosted by a factor *f* in order to incentivise sharing and then divided equally between the players.

Figure 2(d) shows results from a 2-player game, modelled as a CSG. Player choices are necessarily *concurrent*, to avoid cheating. We also need to use *equilibria* since the players have distinct individual goals (maximising personal expected profit). Figure 2(d) shows the values for each player in a synthesised optimal (social welfare Nash) equilibrium for varying *f*. Changes in *f* affect both the resulting profit *and* potential inequalities between players in equilibria, indicating the subtleties involved when tuning parameters in an incentive mechanism and the usefulness of analysing this with PRISM-games.

**Table 1.** Model statistics for some of the case studies.

Case study	Players	States transitions	Constr. time(s)	Property	Verif. time(s)
*Robot* coordination	2	159,202 10,765,010	30.94	$\langle \! \langle {p_1} \rangle \! \rangle {\mathtt P}_{\max =?}[\,{\lnot \mathsf {c} \, {\mathtt {U}^{\leqslant k}}\mathsf {g}_1}\,]$	114.5
	2	159,202 10,765,010	39.00	$\langle \! \langle {p_1{:}p_2} \rangle \! \rangle _{\max =?}({\mathtt P}_{}[\,{\lnot \mathsf {c} \, {\mathtt {U}^{\leqslant k}}\mathsf {g}_1}\,]{+}{\mathtt P}_{}[\,{\lnot \mathsf {c} \, {\mathtt {U}^{\leqslant k}}\, \mathsf {g}_2}\,])$	1,080
*Future markets* investors	3	1,398,441 7,374,616	51.2	$\langle \! \langle {i_1} \rangle \! \rangle {\mathtt R}^{}_{\max =?}[\,{{\mathtt {F}^c\ }\mathsf {cashed}_1}\,]$	1,030
	3	478,761 2,265,560	13.47	$\langle \! \langle {i_1{:}i_2} \rangle \! \rangle _{\max =?}({\mathtt R}^{}_{}[\,{{\mathtt {F}\,}\, \mathsf {c}_1}\,]{+}{\mathtt R}^{}_{}[\,{{\mathtt {F}\,}\, \mathsf {c}_2}\,])$	13,110
*User-centric* networks	7	2,993,308 11,392,196	198.6	$\langle \! \langle { user } \rangle \! \rangle {\mathtt R}^{}_{\max =?}[\,{{\mathtt {F}^c\ } services {=}K}\,]$	1,061
*Aloha*	3	556,168 2,401,113	15.7	$\langle \! \langle {p_2,p_3} \rangle \! \rangle {\mathtt R}^{}_{\min =?}[\,{{\mathtt {F}\,}\, \mathsf {sent}_{2,3}}\,]$	317.8
	3	3,334,681 17,834,254	146.1	$\langle \! \langle {p_1{:}p_2{,}p_3} \rangle \! \rangle _{\min =?}({\mathtt R}^{}_{}[\,{{\mathtt {F}\,}\, \mathsf {s}_1}\,]{+}{\mathtt R}^{}_{}[\,{{\mathtt {F}\,}\, \mathsf {s}_{2,3}}\,])$	3,129
*Task graph* scheduling	2	659,948 1,798,198	11.16	$\langle \! \langle { sched } \rangle \! \rangle {\mathtt R}^{}_{\max =?}[\,{{\mathtt {F}\ } done }\,]$	89.7

**Scalability and Performance.** Finally, we show some experimental results for a representative selection of larger examples, to give an indication of the scalability and performance of PRISM-games 3.0. Table 1 shows a range of models (the first 4 are CSGs; the last is a TPTG), the statistics for each one (number of players, states, transitions) and the time taken to build and verify the model for some example properties on a 2.10 GHz Intel Xeon with 8 GB of JVM memory.

Verification of CSGs is more computationally expensive than for TSGs supported in earlier versions of the tool, but PRISM-games 3.0 is able to build and analyse CSGs with more than 3 million states on relatively modest hardware. The majority of the time is spent solving (bi)matrix games, which is done repeatedly for all states of the model. Hence, the number of choices per state, which dictates the size of these games, has a greater impact on performance than for TSGs. Unsurprisingly, equilibria properties are slower than zero-sum ones. For both types of property, the number of players in the game does not have a major impact since they are grouped into coalitions yielding a 2-player game to solve. For TPTGs, the digital clocks translation is fast since it is done syntactically, and then a TSG is solved whose size depends on several factors, primarily the number of locations and the magnitude of any time bound in the property.

## Conclusions

We have presented PRISM-games 3.0, which adds three major new features: (i) concurrent stochastic games; (ii) synthesis of equilibria; and (iii) timed probabilistic games. The usefulness of these has been illustrated on several newly created or extended applications.

CSGs are considerably more expensive to solve than their turn-based counterparts and a key challenge is efficiently solving the matrix game at each state, which is itself a non-trivial optimisation problem. For equilibria, the main difficulty is finding an optimal equilibrium, which currently relies on iteratively restricting the solution search space. Both problems are sensitive to the limitations and issues of floating-point arithmetic, particularly equilibria computation, and might benefit from arbitrary precision representations. Recent research has also pointed out the shortcomings of only using a lower bound approximation as a stopping criterion for value iteration, as it can lead to inaccuracies [4, 14, 17]. The impact of similar issues on model checking for games is still to be studied.

A range of further challenges exist for future work. These include providing support for *multi-coalitional* properties and implementing other techniques for equilibria computation. For timed games, we plan to investigate concurrent variants, and also zone-based solution techniques. More broadly speaking, partial information variants of games would be a useful addition.
